# Case II. Rheumatic Inflammation of Spinal Nerves

**Published:** 1871-02

**Authors:** 


Case II—Rheumatic Inflammation of Spinal Nerves.—This patient has been complaining a considerable length of time, with symptoms resembling those of ordinary rheumatism, but differing from them in some respects. Was admitted a week ago, complaining of a troublesome cough. Says he takes cold easily, which is followed by a hard, tight cough that annoys him more in the latter part of the night, and is very harrassing. His chest is free from dulness or any abnormal sound; pulse of natural frequency and force; temperature of the skin natural.

Aside from the cough, and a vague sense of soreness in the chest, there is a degree of lameness in the hip, manifesting itself in a manner somewhat different from simple muscular stiffness.

In the act of rising from the sitting posture there is frequently great and sudden, spasmodic contraction of certain muscles, more especially in the right hip; that brings him down. Does not complain of any sharp pain running along the course of the sciatic nerve, as in rheumatism, involving the origin of this nerve. The pain appears to be in the gluteal and abductor muscles. He locates the pain in both hips, but mostly in the right; there is some tenderness in the bottom of the foot on bearing his weight upon it, and some morbid sensibility of the parts.

The cramp and an alteration in his gait raised the question whether this was a case of simple rheumatic inflammation, involving the muscles, or whether he was laboring under disease of the spinal cord, constituting the early stage of that form of disease styled progressive locomotor ataxia. But in watching his movements, and noting the changeable character of the pain, florid countenance and sanguine temperament, the Professor was led to conclude that there was no tendency to atrophy of the spinal cord; but true rheumatic inflammation, involving certain portions of the cord that supply the parts in which he complains of pain. There is probably the same grade of inflammation involving the fibrous structure of the bronchial tubes, which gives rise to the cough.

The diagnosis of these cases requires great care. The early stage of hip disease is often mistaken for rheumatism, till the parts begin to fill up with matter; and the same is true of inflammation in the psoas region.

In hip disease, however, if you will place the patient upon the back, with your hands upon the trochanters, and press the head of the femur into the socket; he will complain at once of pain. In addition to this, if you take hold of the foot, straighten the leg, and push up, you will produce the same result. Rheumatism, on the contrary, will not be affected by such pressure; at least it will produce only superficial pain, and it will hurt more to pull the hip out than pushing it back. In the early stage of hip disease, also, the toe is turned in and the heel drawn up.

If the affection is in the psoas region, the thigh is more or less flexed, and the patient is unable te extend it.

In this case we have absence of all of these conditions, and hence regard the patient as laboring under simple chronic rheumatic inflammation. On admission to the hospital, he was put upon the following treatment:

℞ Vin. Colch. Sem., ℥j.

Tinct. Aconit. Rad., ℨj.

Tinct. Strammonii, ℥ss.

Syrup and water, ℥iiss.

M.

Dose, one teaspoonful every four hours.

To procure more rest at night, he took 15 grains of potassii bromide at bedtime.

He has been under this treatment one week, and his condition is very much ameliorated; the colchicum has not disturbed the bowels; the rheumatic trouble about the hip and back is improved; he has no muscular cramps, and goes about tolerably free.

In those cases of rheumatic inflammation chiefly restricted to nerve structure, the Professor stated that he had not found ordinary alkaline salts to produce as satisfactory results as some other modes of treatment, and had found it necessary to use remedies that have a more powerfully sedative influence upon the nervous sensibility.

There appears to be irritation set up in the nerve structure, which the alkaline salts alone are not able to overcome.

He had found the above combination, in patients of sanguine temperament, to be very valuable.

In persons less sanguinous, and with a less degree of excitability of the circulation, he would leave out the aconite, which is added to increase the sedative effect upon the nervous system.

The same treatment was continued another week, when the patient was discharged, quite well.

